# A quantitative approach to nucleophilic organocatalysis

**DOI:** 10.3762/bjoc.8.166

**Published:** 2012-09-05

**Authors:** Herbert Mayr, Sami Lakhdar, Biplab Maji, Armin R Ofial

**Affiliations:** 1 Department Chemie, Ludwig-Maximilians-Universität München, Butenandstr. 5-13 (Haus F), 81377 München, Germany

**Keywords:** enamine activation, iminium activation, kinetics, NHC activation, organocatalysis, structure reactivity relationships

## Abstract

The key steps in most organocatalytic cyclizations are the reactions of electrophiles with nucleophiles. Their rates can be calculated by the linear free-energy relationship log *k*(20 °C) = *s*_N_(*E* + *N*), where electrophiles are characterized by one parameter (*E*) and nucleophiles are characterized by the solvent-dependent nucleophilicity (*N*) and sensitivity (*s*_N_) parameters.

Electrophilicity parameters in the range –10 < *E* < –5 were determined for iminium ions derived from cinnamaldehyde and common organocatalysts, such as pyrrolidines and imidazolidinones, by studying the rates of their reactions with reference nucleophiles. Iminium activated reactions of α,β-unsaturated aldehydes can, therefore, be expected to proceed with nucleophiles of 2 < *N* < 14, because such nucleophiles are strong enough to react with iminium ions but weak enough not to react with their precursor aldehydes. With the *N* parameters of enamines derived from phenylacetaldehyde and MacMillan’s imidazolidinones one can rationalize why only strong electrophiles, such as stabilized carbenium ions (–8 < *E* < –2) or hexachlorocyclohexadienone (*E* = –6.75), are suitable electrophiles for enamine activated reactions with imidazolidinones. Several mechanistic controversies concerning iminium and enamine activated reactions could thus be settled by studying the reactivities of independently synthesized intermediates.

Kinetic investigations of the reactions of N-heterocyclic carbenes (NHCs) with benzhydrylium ions showed that they have similar nucleophilicities to common organocatalysts (e.g., PPh_3_, DMAP, DABCO) but are much stronger (100–200 kJ mol^–1^) Lewis bases. While structurally analogous imidazolylidenes and imidazolidinylidenes have comparable nucleophilicities and Lewis basicities, the corresponding deoxy Breslow intermediates differ dramatically in reactivity. The thousand-fold higher nucleophilicity of 2-benzylidene-imidazoline relative to 2-benzylidene-imidazolidine is explained by the gain of aromaticity during electrophilic additions to the imidazoline derivatives. O-Methylated Breslow intermediates are a hundred-fold less nucleophilic than deoxy Breslow intermediates.

## Review

### Introduction

The most comprehensive nucleophilicity and electrophilicity scales presently available, are based on Equation 1, in which electrophiles are characterized by one solvent-independent parameter *E,* and nucleophiles are characterized by two solvent-dependent parameters, the nucleophilicity parameter *N* and the sensitivity parameter *s*_N_ [1–3].

[1]
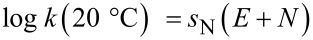


**Figure 1:**
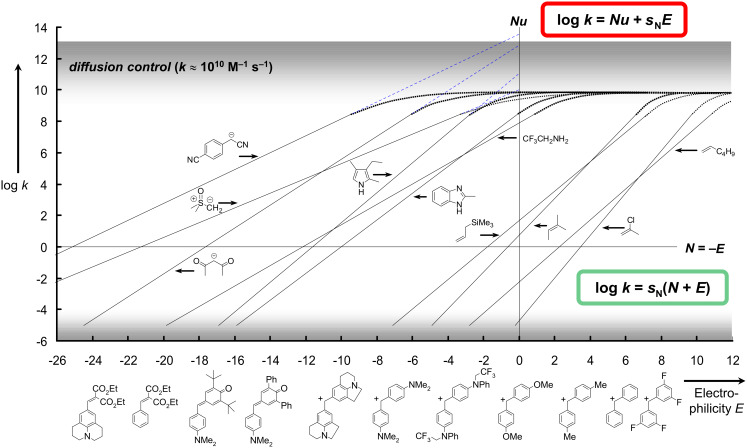
Second-order rate constants for reactions of electrophiles with nucleophiles.

By defining benzhydrylium ions, structurally related quinone methides, and arylidenemalonates as reference electrophiles, which cover a reactivity range of 32 orders of magnitude corresponding to relative reaction times from nanoseconds to 10^15^ years, we have been able to compare nucleophiles of widely differing structure and reactivity [4]. As illustrated by Figure 1, this method allows us to characterize strong nucleophiles, such as carbanions and ylides, by their reactivities toward weak electrophiles, and to characterize weak nucleophiles, such as nonactivated alkenes, by their reactivities toward strong electrophiles. Recently we have explicitly outlined the reasons why we prefer Equation 1, a nonconventional version of a linear free-energy relationship, which defines nucleophilicities as the negative intercepts on the abscissa, over the conventional (mathematically equivalent) linear free-energy relationship depicted in the red frame at the top of Figure 1 [5].

The reactivity scales, developed on this basis, have not only be employed for designing organic syntheses [6–18], but were also helpful for rigorous examinations of general concepts of organic reactivity, such as the “Reactivity Selectivity Principle” [19], the “HSAB Treatment of Ambident Reactivity” [20] and the changes of mechanisms in nucleophilic aliphatic substitutions [21–22]. In this essay, we will illustrate applications of Equation 1 in nucleophilic organocatalysis.

### Iminium activated reactions

**Figure 2:**
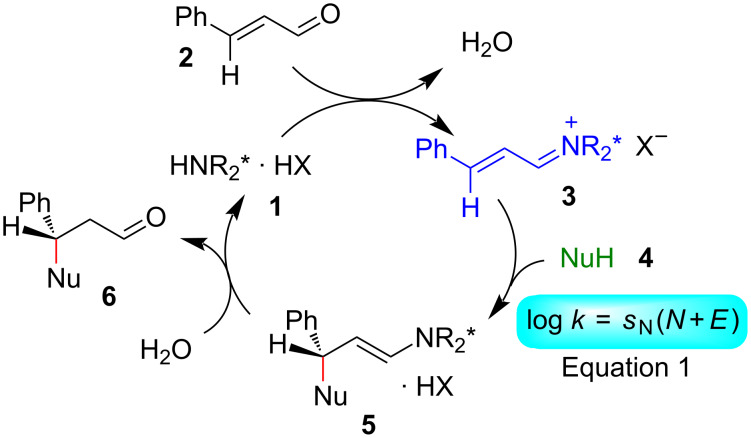
Mechanism of amine-catalyzed conjugate additions of nucleophiles [23–28].

A key step of the commonly accepted catalytic cycle for iminium activated reactions (Figure 2) is the attack of a nucleophile **4** on the intermediate iminium ion (**3**), which can be treated by Equation 1 as indicated in the bottom right of Figure 2 [23–28].

In order to predict which nucleophiles **4** are suitable reagents for such transformations because they are strong enough to react with iminium ions **3**, but weak enough not to react with the precursor carbonyl compounds (e.g., **2**), it was necessary to determine the reactivity parameters *N* and *s*_N_ of potential nucleophilic substrates **4** and the electrophilicity parameters *E* of iminium ions **3**.

**Figure 3:**
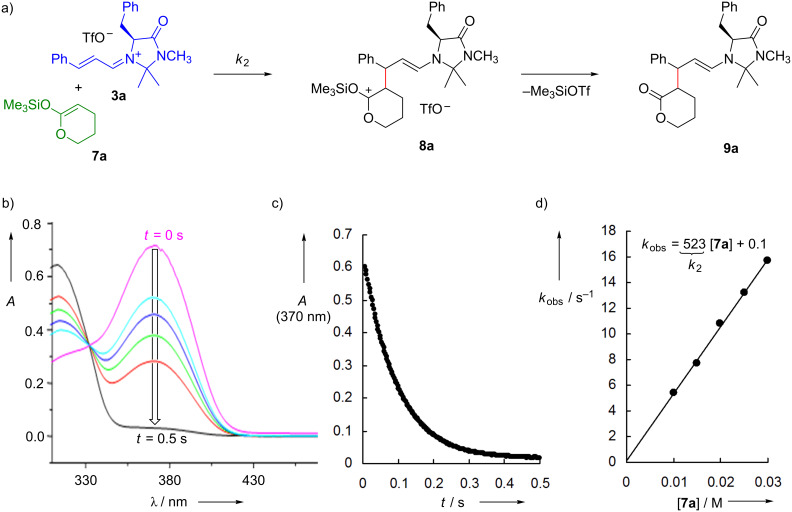
Kinetics of the reactions of the iminium ion **3a** with the silylated ketene acetal **7a** [35].

Iminium triflates, tetrafluoroborates, or hexafluorophosphates were synthesized as stable salts according to literature procedures [29–35]. Cinnamaldehyde-derived iminium ions **3** are particularly suitable for kinetic investigations because their reactions with nucleophiles can easily be followed photometrically by monitoring the decay of their absorbance at 370 nm (as exemplified in Figure 3a,b). By using the nucleophiles (for example **7a**) in large excess, pseudo-first-order kinetics were achieved, and the first-order rate constants *k*_obs_ (s^–1^) were derived from the exponential decays of the iminium ions **3** (Figure 3c). Plots of *k*_obs_ versus the concentrations of the nucleophiles (Figure 3d) were linear, with their slopes giving the second-order rate constants *k*_2_ (M^–1^ s^–1^) [35–36].

**Figure 4:**
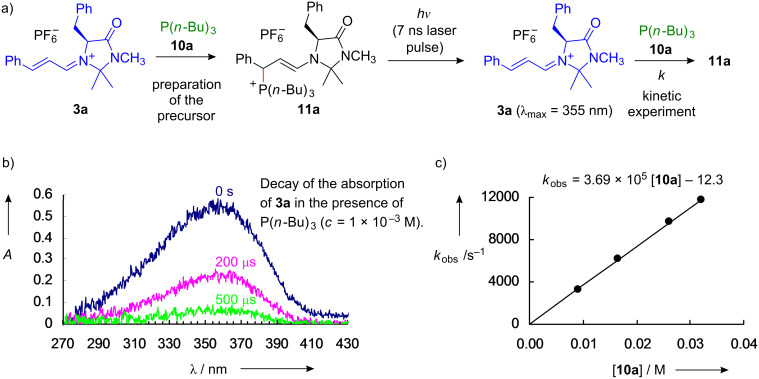
Laser flash photolytic generation of iminium ions **3a**.

For the investigations of reactions of the iminium ions on the micro- and nanosecond time scale, laser flash spectroscopy was employed [37]. As tertiary phosphines PR_3_ (**10**) are known to be excellent photonucleofuges [38–41], the stable iminium salts **3**-PF_6_ were treated with tertiary phosphines **10** at room temperature to give the enamino-phosphonium ions **11** instantaneously (Figure 4a). Their irradiation with 7 ns laser pulses (266 nm) regenerated the iminium ions, the decay of which was monitored photometrically in the presence of variable concentrations of nucleophiles (Figure 4b).

As above, the second-order rate constants for the reactions of the iminium ions with nucleophiles were obtained as the slopes of the plots of the first-order rate constants *k*_obs_ versus the concentrations of the corresponding nucleophiles (Figure 4c).

**Figure 5:**
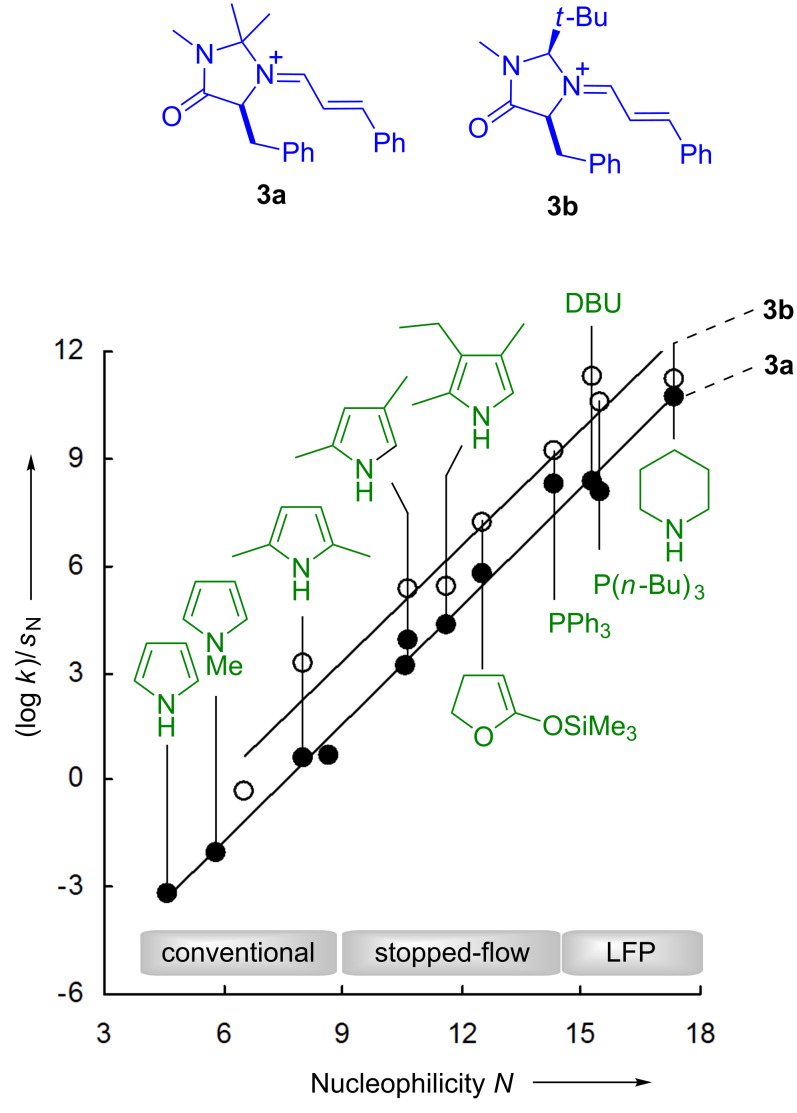
Correlations of the reactivities of the iminium ions **3a** and **3b** toward nucleophiles with the corresponding *N* parameters – LFP = laser flash photolysis.

The fair correlations of (log *k*_2_)/*s*_N_ versus the nucleophilicity parameters *N* with slopes of unity in Figure 5 indicate the applicability of Equation 1, and is a further evidence that the reactivity parameters *N* and *s*_N_, which are derived from reactions with benzhydrylium ions, also hold for reactions with iminium ions **3**.

**Figure 6:**
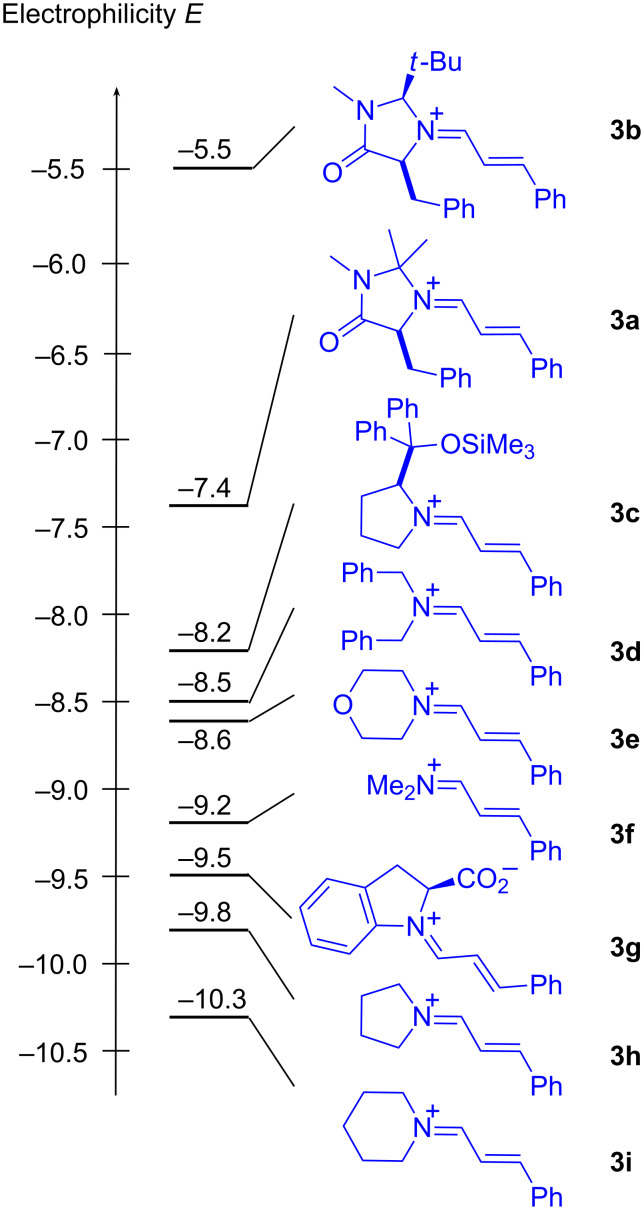
Comparison of the electrophilicities of cinnamaldehyde-derived iminium ions **3a**–**3i**.

Analogous experiments showed that the cinnamaldehyde-derived iminium ions **3a**–**i** cover a reactivity range of five orders of magnitude; the iminium ion **3b**, derived from MacMillan’s generation II catalyst, turned out to be by far the most reactive one of this series (Figure 6) [37,42–43].

**Figure 7:**
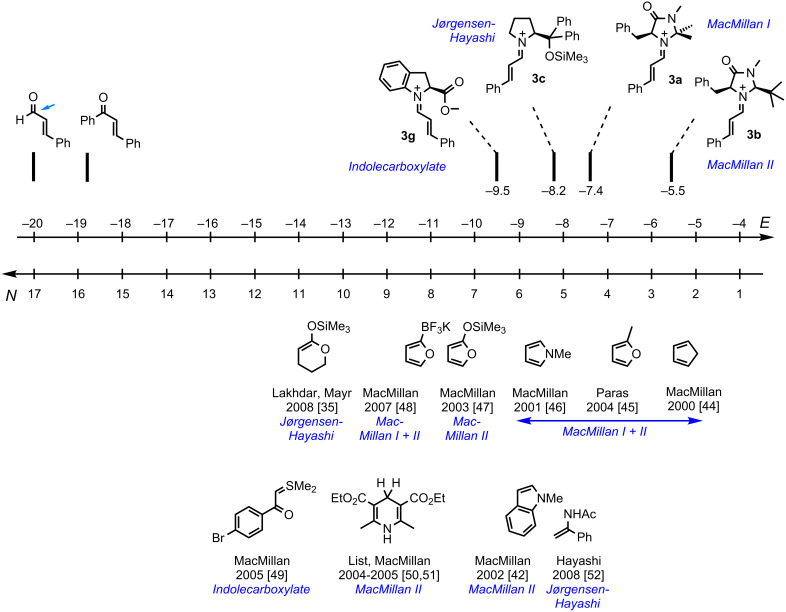
Nucleophiles used in iminium activated reactions [35,42,44–52].

When comparing the *N* parameters of substrates previously employed in iminium activated reactions (Figure 7) [35,42,44–52], one can see that they are characterized by nucleophilicity parameters in the range 2 < *N* < 14. As Equation 1 describes only one step of the catalytic cycle in Figure 2, we do not claim that *N* parameters in the indicated range represent a sufficient criterion for the selection of potential substrates in iminium activated reactions. It will be difficult, however, to find suitable nucleophilic substrates outside this range, as stronger nucleophiles will either react with the carbonyl compounds directly or inhibit the formation of the iminium ions due to their high basicity. Weaker nucleophiles, on the other hand, will not be able to attack iminium ions **3**; exceptions may be expected for substrates which undergo concerted pericyclic reactions with the iminium ions and therefore do not follow Equation 1 [53].

Let us now consider the role of counterions, as the imidazolidinone catalyzed reactions of cinnamaldehyde with pyrrole were reported to proceed with high yields and enantioselectivities, when using trifluoroacetic acid as cocatalyst, while yields and enantioselectivities are low with strong acids, such as CF_3_SO_3_H, TsOH, or HCl, as cocatalysts [46,54–55].

**Figure 8:**
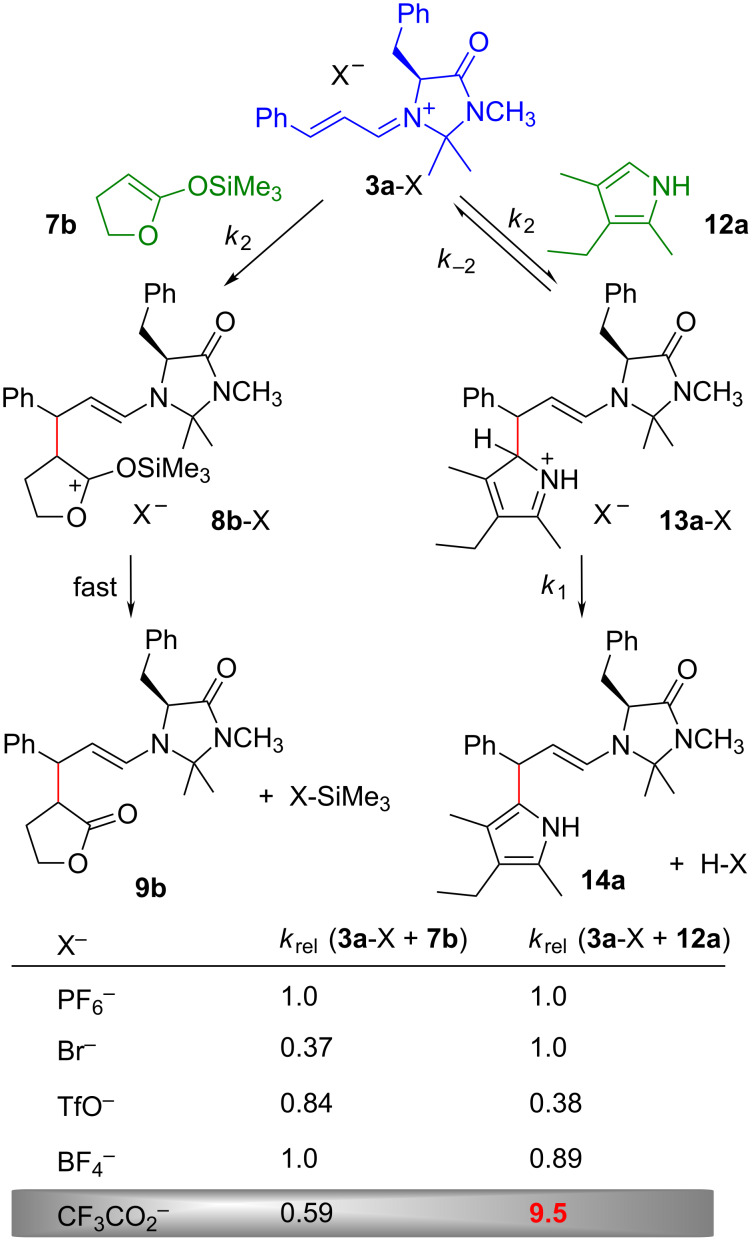
Counterion effects in electrophilic reactions of iminium ions **3a**-X (at 20 °C, silyl ketene acetal **7b** in dichloromethane with *c*(**3a**-CF_3_CO_2_) = (1.7–2.5) × 10^−5^ M, kryptopyrrole **12a** in acetonitrile with *c*(**3a**-CF_3_CO_2_) = 5.0 × 10^−5^ M).

Figure 8 shows that the rates of the reactions of **3a**-X with 2-(trimethylsiloxy)-4,5-dihydrofuran (**7b**) were only slightly affected by the nature of the counterions X^–^ (X^–^ = PF_6_^–^, BF_4_^–^, TfO^–^, Br^–^, CF_3_CO_2_^–^) [56].

**Figure 9:**
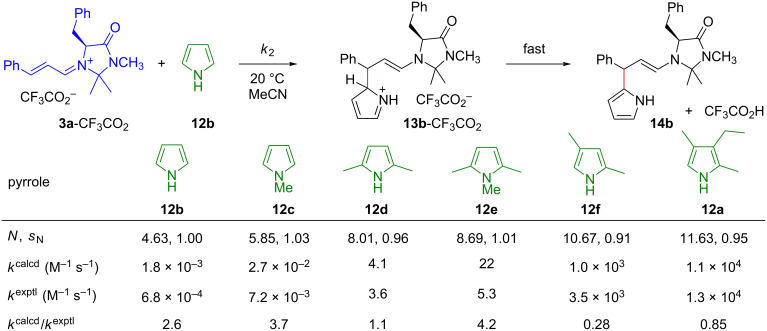
Comparison of calculated and experimental rate constants of electrophilic aromatic substitutions with iminium ions [56].

In contrast, the reaction of **3a**-X with 3-ethyl-2,4-dimethylpyrrole (kryptopyrrole, **12a**) was considerably faster when CF_3_CO_2_^−^ was present than when less basic counterions were employed. The acceleration of the reaction by increasing the concentration of CF_3_CO_2_^–^ demonstrated that CF_3_CO_2_^–^ acted as a general base to deprotonate the Wheland intermediate **13a^+^** and thus suppresses its retroaddition with regeneration of the pyrrole **12a** and the iminium ion **3a**. Rate constants observed at variable concentrations of CF_3_CO_2_^–^ allowed us to calculate the second-order rate constants *k*_2_ for the attack of the iminium ion **3a** at the pyrroles **12a**–**12f**, and Figure 9 shows that the observed rate constants agree, within a factor of five, with those calculated by using Equation 1.

We consider this agreement remarkable, as the *E* parameter for **3a** has been derived from rate constants with a large variety of nucleophiles [37] and the *N* and *s*_N_ parameters of the pyrroles **12a**–**12f** have been derived from their reactivities toward benzhydrylium ions [57]. As Equation 1 is employed for calculating absolute rate constants *k*_2_ in a reactivity range of 40 orders of magnitude with only three parameters, *N*, *s*_N_, and *E*, one generally has to tolerate deviations up to factors of 10 to 100 [2–3,5].

**Figure 10:**
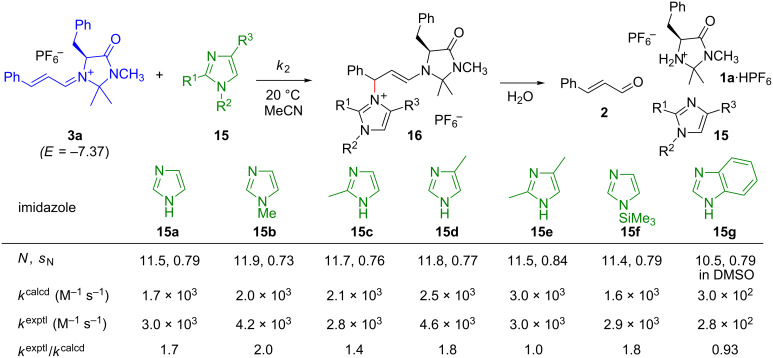
Aza-Michael additions of the imidazoles **15** with the iminium ion **3a** [58].

However, an even better agreement between calculated and experimental values was observed for the reactions of **3a** with imidazoles **15** (Figure 10) [58].

These additions are highly reversible, however, and the adducts could only be isolated when the reaction mixtures containing **16** (for R^2^ = H) were worked up with dry K_2_CO_3_. Aqueous workup led to regeneration of the reactants. Vicario’s report that imidazoles, in contrast to triazoles and tetrazoles, do not readily undergo iminium activated additions to α,β-unsaturated aldehydes can thus be explained by the low acidity of imidazolium ions [59]. Unlike triazolium and tetrazolium ions, imidazolium ions are unable to transfer a proton to the enamine unit in **16** (corresponding to **5** in the general Figure 2), which is necessary to close the catalytic cycle shown in Figure 2 [60].

**Figure 11:**
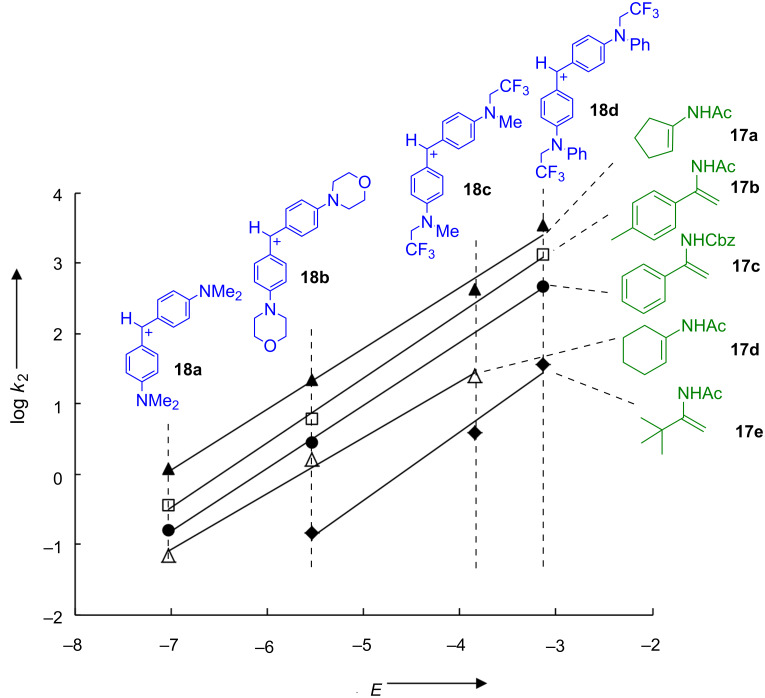
Plots of log *k*_2_ for the reactions of enamides **17a**–**17e** with the benzhydrylium ions **18a–d** in CH_3_CN at 20 °C versus the electrophilicity parameters (*E*).

**Figure 12:**
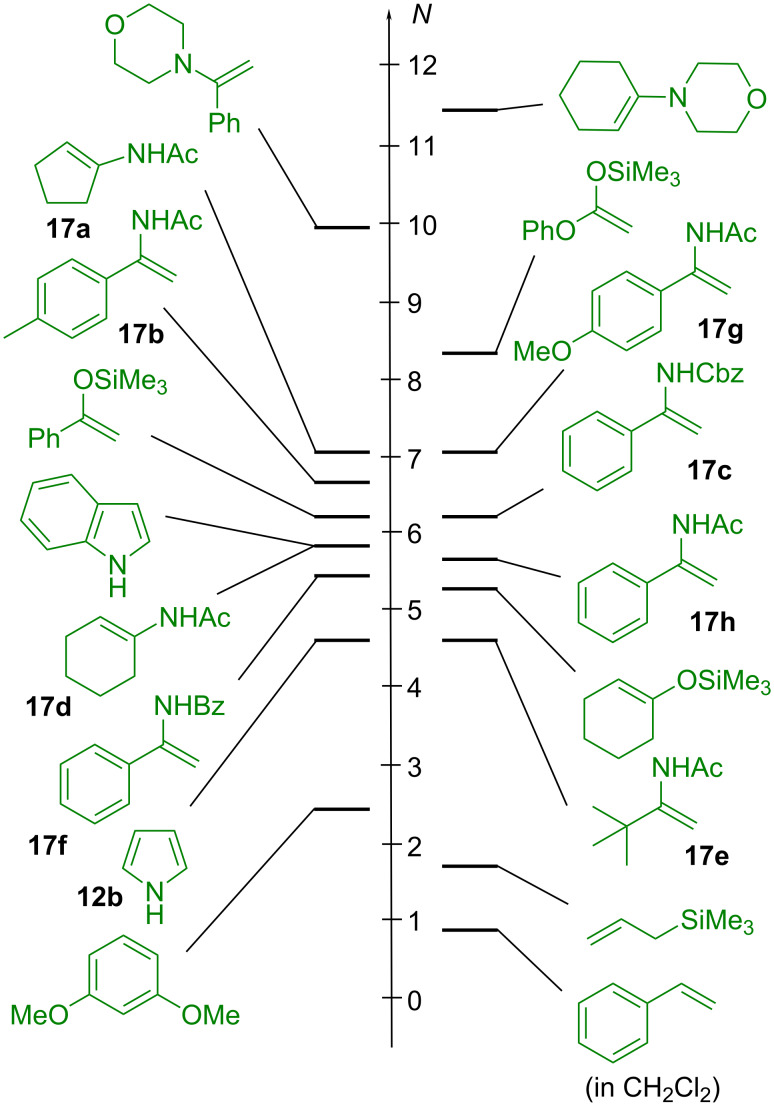
Comparison of the nucleophilicities of enamides **17** with those of several other C nucleophiles (solvent is CH_3_CN unless otherwise mentioned, *N* values taken from [4,61]).

General base catalysis appeared also to be essential for iminium activated reactions of α,β-unsaturated aldehydes with enamides **17**. By studying the kinetics of the reactions of enamides **17** with benzhydrylium ions **18** (Figure 11) we determined the reactivity parameters *N* and *s*_N_ for these π-nucleophiles, which are listed in Figure 12 [61].

Figure 12 shows that the nucleophilicities *N* of the enamides **17** are comparable to those of silylated enol ethers, in between those of allylsilanes and enamines. Accordingly, we expected them to react readily with the iminium ions **3** at room temperature.

**Figure 13:**
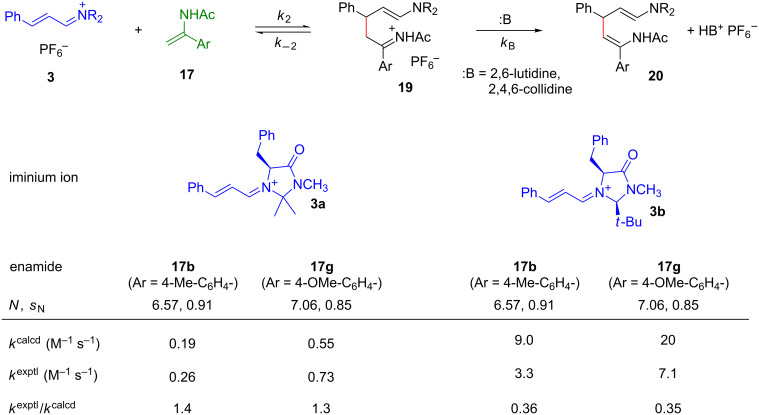
Experimental and calculated rate constants *k*_2_ for the reactions of **17b** and **17g** with **3a** and **3b** in the presence of 2,6-lutidine in CH_2_Cl_2_ at 20 °C [61].

However, when the iminium triflates or hexafluorophosphates **3a** and **3b** (~ 5 × 10^–5^ M) were combined with 25 equivalents of the enamides **17b** and **17g** in CH_2_Cl_2_ or CH_3_CN, no consumption of the iminium ions was observed [61]. These reactions took place in the presence of 2,6-lutidine, however, indicating the need of general base assistance. By studying the kinetics of these reactions in the presence of variable concentrations of 2,6-lutidine, we were able to determine *k*_2_, the rate constant for the attack of the iminium ions **3** at the enamides **17**. As shown in Figure 13, the rate constants thus determined, agree within a factor of 3 with those calculated by Equation 1 using the *N* and *s*_N_ parameters of enamides **17**, which have been derived from their reactions with the benzhydrylium ions **18** (Figure 11 and Figure 12) [61].

These observations explain why strong acids, such as *p*-TsOH, proved not to be suitable cocatalysts for iminium activated reactions of α,β-unsaturated aldehydes with enamides [62]. The demonstration of general base catalysis for these reactions furthermore rules out Hayashi’s proposal of a concerted ene reaction for the formation of tetrahydropyridines by the diphenylprolinol-catalyzed reaction of α,β-unsaturated aldehydes with enamides [52] and is in line with Wang’s stepwise mechanism with initial formation of **19** [62].

**Figure 14:**
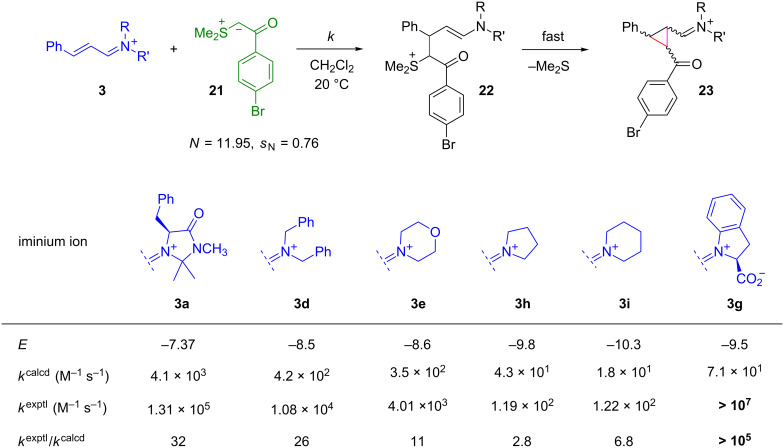
Comparison between experimental and calculated (Equation 1) cyclopropanation rate constants [64].

In view of the high nucleophilicities of sulfur ylides [63], we were surprised by MacMillan’s statement that iminium ions derived from the imidazolidinones **1a** and **1b** (for structures, see Figure 16) were inert to the ylide **21** [49]. When we combined the pregenerated iminium salts **3a**–**e** with the sulfur ylide **21**, the expected cyclopropanes **23** were indeed formed in good yield, although with low diastereo- and enantioselectivity (Figure 14) [64].

**Figure 15:**
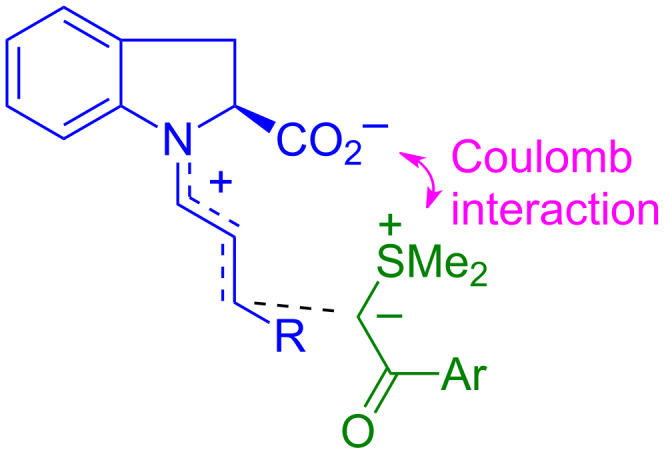
Electrostatic activation of iminium activated cyclopropanations with sulfur ylides.

Even the rate constants calculated by Equation 1 agreed, within the general tolerance, with the experimental values; with one exception. The iminium intermediate derived from indole-2-carboxylic acid (**3g**) reacted at least 10^5^ times faster with the sulfur ylide **21** than calculated by Equation 1, which can be explained by electrostatic activation as initially proposed by MacMillan (Figure 15) [49].

**Figure 16:**
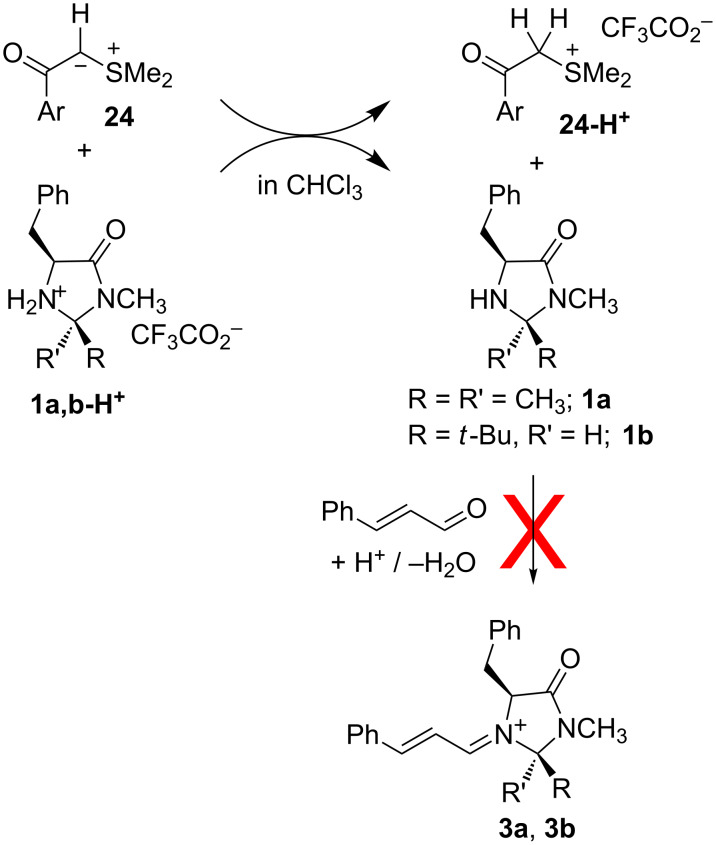
Sulfur ylides inhibit the formation of iminium ions.

Thus, the failure of the imidazolidinones **1a** and **1b** to catalyze cyclopropanations with the sulfur ylide **21** is not due to the low reactivities of sulfur ylides toward iminium ions, but is due to the high Brønsted basicity of the sulfur ylides **24**, which leads to deprotonation of the imidazolidinium ions **1**-H^+^ and inhibition of the formation of the iminium ions **3** (Figure 16) [64].

### Enamine activated reactions

**Figure 17:**
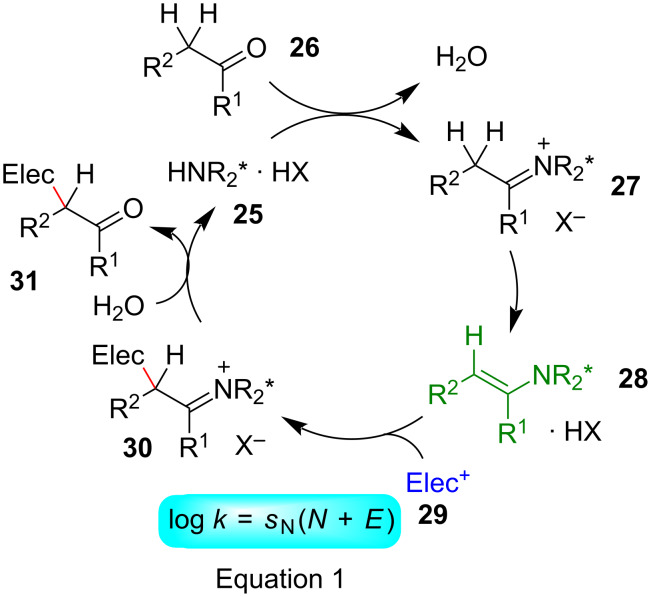
Enamine activation [65].

When proline catalysis and related amino-acid catalyzed reactions are excluded, the catalytic cycle depicted in Figure 17 represents the generally accepted mechanism for enamine activated reactions [65–71]. A key-step, not necessarily the rate-determining step, is the attack of an electrophile **29** at the enamine **28**, at the bottom of Figure 17 [72].

In order to calculate the rate constant for this step by Equation 1 one needs the reactivity parameters *N* and *s*_N_ for the enamines **28** and the electrophilicity parameter *E* for the electrophiles **29**.

**Figure 18:**
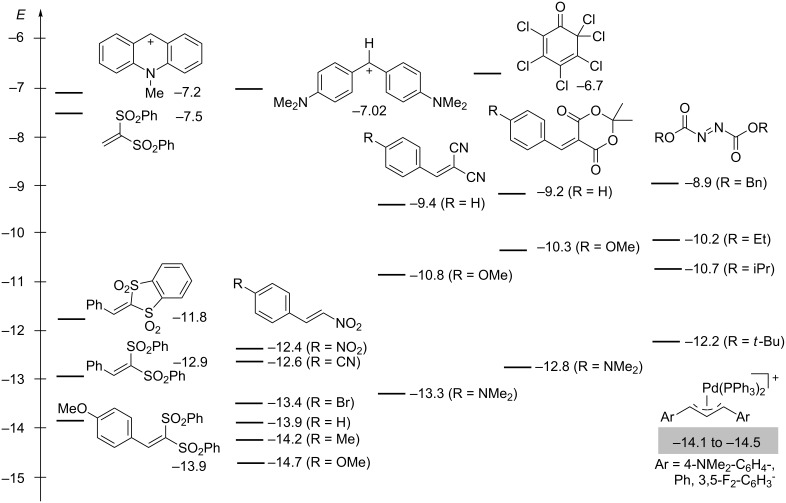
Electrophilicity parameters *E* for classes of compounds that have been used as electrophilic substrates in enamine activated reactions [4,73–80].

The electrophilicity parameters for the Michael acceptors, stabilized carbenium ions, and azodicarboxylates shown in Figure 18 have been derived from the kinetics of their reactions with C-nucleophiles, mostly stabilized carbanions [4,73–80].

**Figure 19:**
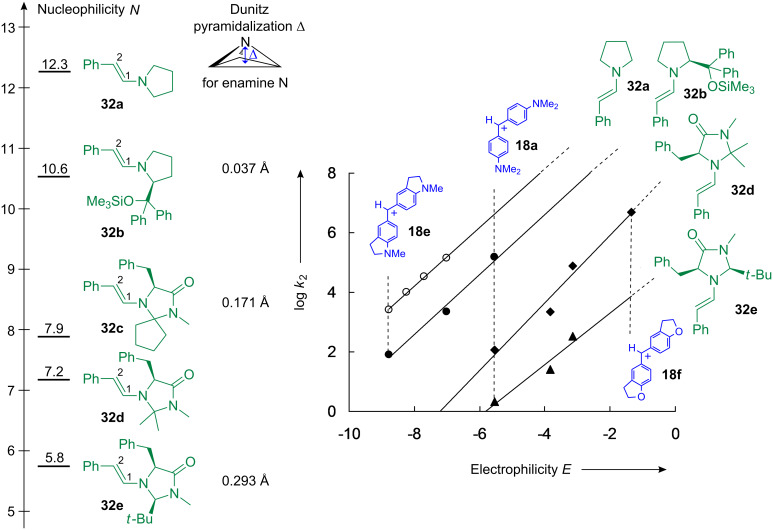
Quantification of the nucleophilic reactivities of the enamines **32a**–**e** in acetonitrile (20 °C) [83]; a definition of the Dunitz pyramidalization Δ is given in [84].

As illustrated in Figure 19, the benzhydrylium methodology was again employed for the determination of the nucleophilicities of enamines. Whereas the enamine **32b**, which is derived from the diphenylprolinol silyl ether [81], had previously been synthesized and characterized (X-ray structure) by Seebach et al. [30], neat samples of the imidazolidinone-derived enamines **32c**–**32e** became only recently available by TsOH-catalyzed condensation of phenylacetaldehyde with the corresponding imidazolidinones and column chromatography on silica gel. The presence of triethylamine (5%) in the eluent (ethyl acetate/*n*-pentane) turned out to be crucial to avoid decomposition of these enamines on the column [82–83].

Kinetic studies of their reactions with benzhydrylium ions **18** of suitable electrophilicity showed that introduction of the (Me_3_SiO)Ph_2_C-group in the 2-position of the pyrrolidine ring of *N*-(β-styryl)pyrrolidine caused a reduction of reactivity by a factor of 30 to 60 (**32a** versus **32b**). A reduction of nucleophilicity by three to five orders of magnitude is encountered for the enamines **32c**–**32e** (Figure 19). The low nucleophilicities of the imidazolidinone derived enamines, which are in line with the larger ^13^C NMR chemical shifts of C-2 in **32d** (101.9 ppm) and **32e** (102.9 ppm) compared to that of C-2 in **32a** (97.4 ppm), are not only due to the electron-withdrawing effect of the additional heteroatoms in the heterocyclic rings [83]. An additional factor is shown in Figure 19: While the enamine nitrogen is almost planar in **32b**, it becomes pyramidalized in the enamines **32c** and **32e** and thus has a weaker electron-donating effect because of the reduced overlap between the nitrogen lone-pair and the π_C–C_-bond.

Combination of the data in Figure 18 and Figure 19 now explains why the Jørgensen-Hayashi diphenylprolinol trimethylsilyl ether [81], the precursor of **32b**, and structurally related pyrrolidines have previously been employed for catalyzing the reactions of aldehydes and ketones with weak electrophiles, such as β-nitrostyrene (*E* = –13.9) [85] or di-*tert*-butyl azodicarboxylate (*E* = –12.2) [86]. The less basic imidazolidinones, which yield the less nucleophilic enamines **32d** and **32e**, are suitable catalysts for reactions with stronger electrophiles, such as the chlorinating agent 2,3,4,5,6,6-hexachlorocyclohexan-2,4-dien-1-one (*E* = –6.75) [87] and, in particular, stabilized carbocations, which are generated in situ from the corresponding alcohols under weakly acidic conditions [14,88–89]. Suggestions for further promising electrophilic reaction partners in enamine activated reactions [90] can be derived from the electrophilicity scales in [4].

**Figure 20:**
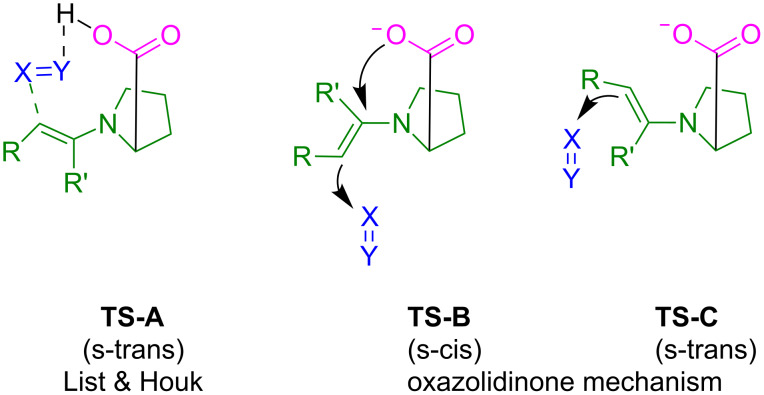
Proposed transition states for the stereogenic step in proline-catalyzed reactions.

When proline or structurally related bifunctional catalysts are employed, the mechanism depicted in Figure 17 has to be modified. List and Houk explained the high enantioselectivity of proline catalyzed reactions of aldehydes or ketones with electrophiles by the transition state **TS-A** in Figure 20, in which the electrophile is activated by the proton of the carboxy group [71]. The formation of oxazolidinones, the only observable intermediates of this reaction cascade, was considered to be an unproductive dead end [70]. On the other hand, Seebach and Eschenmoser raised the question of whether oxazolidinones, rather than being “parasitic species”, may also play a decisive role in determining the stereochemical course of proline-catalyzed reactions. In order to account for the observed stereoselectivities, it was suggested that **TS-B** is favored over the stereoelectronically preferred **TS-C**, because it yields the more stable oxazolidinone [91].

**Figure 21:**
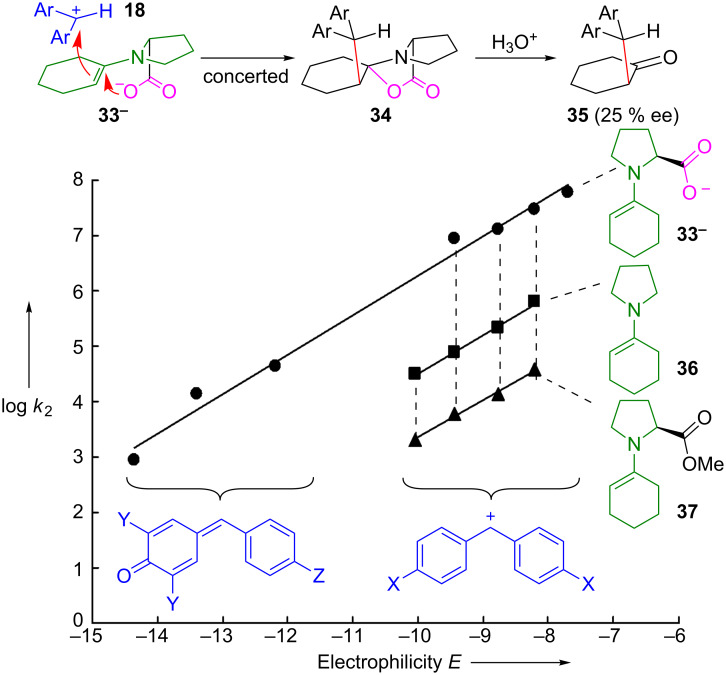
Kinetic evidence for the anchimeric assistance of the electrophilic attack by the carboxylate group*.* The hydrolysis product (*R*)-**35** was obtained with 25% ee from the reaction of **33^−^** (counterion: protonated DBU) with **18a**-BF_4_^−^ (Ar = 4-Me_2_N-C_6_H_4_) in MeCN after aqueous workup [92].

Figure 21 shows that the enaminocarboxylate **33^−^** reacts 50 to 60 times faster with benzhydrylium ions than pyrrolidinocyclohexene **36** and even 800 to 900 times faster than the methyl ester **37** [92].

We consider the high rates of the reactions of **33^−^** with benzhydrylium ions **18** as evidence for anchimeric assistance by the carboxylate group. As only part of the accelerating effect of the CO_2_^−^ group can be due to Coulomb attraction, the formation of the C–O bond of the oxazolidone **34** is concluded to occur concomitantly with the formation of the C–C bond. The observation that β-nitrostyrene, a neutral electrophile, also reacts 10^2^ times faster with **33^–^** than with **36** also excludes Coulomb attraction to be the major factor for the high reactivity of **33^−^**. On the other hand, di-*tert*-butyl azodicarboxylate reacts only six times faster with **33^−^** than with **36**, showing that the magnitude of the anchimeric assistance depends largely on the nature of the electrophile.

The data in Figure 21 thus suggest that the oxazolidinones **34** are formed in the stereodifferentiating step when enaminecarboxylate anions are the effective nucleophiles. However, our observations do not affect the rationalization of the stereoselectivities of proline-catalyzed reactions by **TS-A** when the electrophilic attack occurs at an enaminocarboxylic acid. Blackmond’s observation of a change of enantioselectivity by added bases is in line with our interpretations [93].

### Quantitative aspects of N-heterocyclic carbene (NHC) catalysis

**Figure 22:**
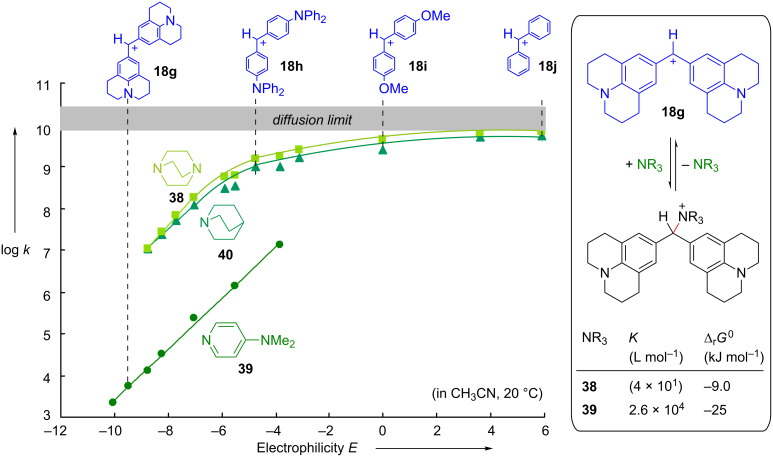
Differentiation of nucleophilicity and Lewis basicity (in acetonitrile at 20 °C): Rate (left) and equilibrium constants (right) for the reactions of amines with benzhydrylium ions [94–95].

As the following discussion will focus on the difference between the kinetic term “nucleophilicity” and the thermodynamic term “Lewis basicity”, let us first illustrate this aspect by comparing the behavior of two well-known organocatalysts, 1,4-diazabicyclo[2.2.2]octane (DABCO, **38**) and (4-dimethylamino)pyridine (DMAP, **39**). As shown in Figure 22, DABCO (**38**) reacts approximately 10^3^ times faster with benzhydrylium ions than DMAP (**39**), i.e., DABCO (**38**) is considerably more nucleophilic than DMAP (**39**) [94].

On the other hand, the equilibrium constant for the formation of the Lewis acid–Lewis base adduct with **18g** is 160 times smaller for DABCO (**38**) than for DMAP (**39**), i.e., DABCO (**38**) is a significantly weaker Lewis base than DMAP (**39**). We have previously discussed that it is the higher reorganization energy for the reaction of DMAP (**39**) that is responsible for the higher intrinsic barrier and subsequently the lower nucleophilicity of DMAP (**39**) [94].

**Figure 23:**
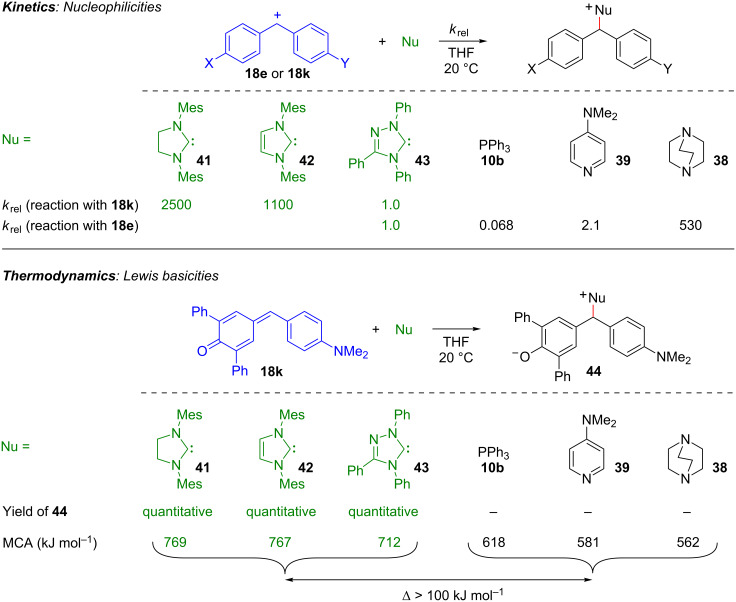
NHCs **41**, **42**, and **43** are moderately active nucleophiles and exceptionally strong Lewis bases (methyl cation affinity, MCA, was calculated for the reaction CH_3_^+^ + Nu → CH_3_–Nu^+^ on MP2/6-31+G(d,p)//B98/6-31G(d) level of theory) [96].

The upper part of Figure 23 compares the relative rates for the reactions of various organocatalysts (in THF) with the benzhydrylium ion **18e** and the structurally related quinone methide **18k**. This comparison reveals that the nucleophilicities of the NHCs **41**–**43** do not differ fundamentally from those of other organocatalysts, e.g., triphenylphosphine (**10b**), DMAP (**39**), and DABCO (**38**) [96].

The considerably lower nucleophilicity of the triazolylidene **43** compared with the imidazolylidene **42** can be explained by the inductive electron withdrawal of the extra nitrogen in the triazol derivative **43**. The similar nucleophilicities of the imidazole- and imidazolidine-derived carbenes **42** and **41** are, at first glance, surprising and will be discussed below. The lower part of Figure 23 illustrates that all three NHCs, **41**, **42**, and **43**, react quantitatively with the quinone methide **18k**, while none of the other Lewis bases, despite their similar nucleophilicities, gives an adduct. The resulting conclusion, that all NHCs are significantly stronger Lewis bases than PPh_3_ (**10b**), DMAP (**39**), and DABCO (**38**), is confirmed by quantum chemical calculations: The methyl cation affinities (MCAs) of the three carbenes **41**–**43** are 100–200 kJ mol^–1^ higher than those of the other Lewis bases in Figure 23 [96].

As the carbenes **41** and **42** have almost identical nucleophilicities and Lewis basicities, the question arose as to why imidazolidine-2-ylidenes (for example, **41**) have rarely been used as organocatalysts, while unsaturated NHCs (for example, **42**) have been reported to catalyze a large variety of reactions [97–104]. Can the difference be explained by the properties of the Breslow intermediates [105]? To address this question, the deoxy Breslow intermediates **45** [106–108] were synthesized by reactions of the NHCs **41**–**43** with benzyl bromides and subsequent deprotonation of the resulting amidinium ions.

**Figure 24:**
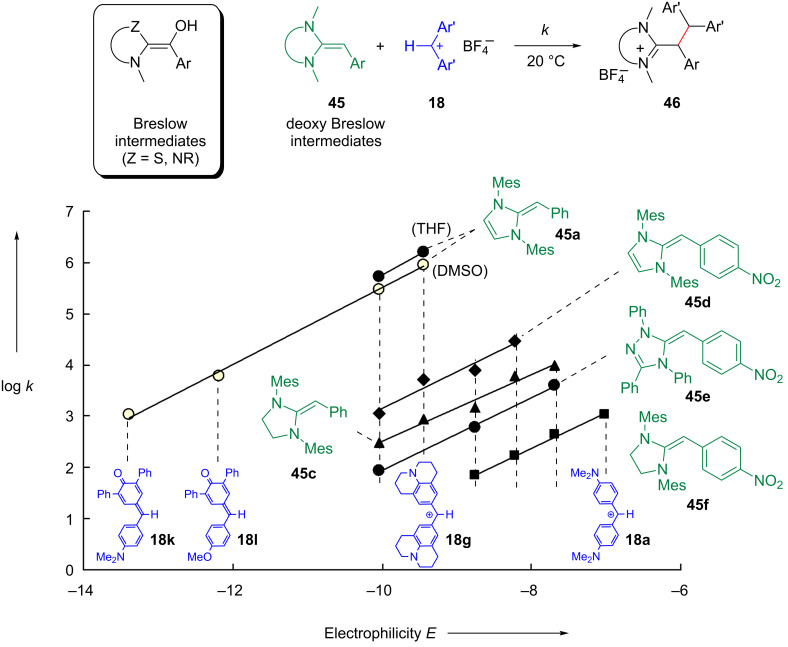
Nucleophilic reactivities of the deoxy Breslow intermediates **45** in THF at 20 °C [107].

**Figure 25:**
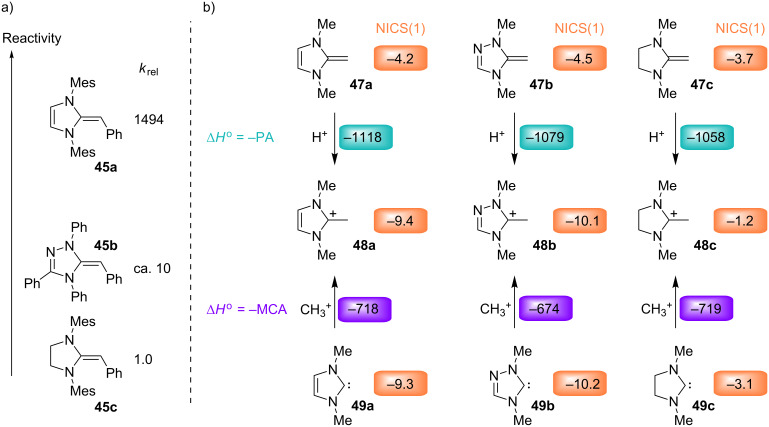
Comparison of the proton affinities (PA, from [107]) of the diaminoethylenes **47a–c** with the methyl cation affinities (MCA, from [96]) of the corresponding carbenes **49a**–**c** (in kJ mol^–1^, MP2/6-31+G(2d,p)//B98/6-31G(d)), and the NICS(1) values of **47**–**49** (B3LYP/6-311+G(d)) (from [107]).

The linear correlations in Figure 24 show that the nucleophilic reactivities of the so-called deoxy Breslow intermediates **45a**–**f** can be described by Equation 1 [107]. In contrast to the situation described for the NHCs in Figure 23, the benzylidene-imidazolines **45a**,**d** are now 10^3^ times more nucleophilic than the corresponding benzylidene-imidazolidines **45c**,**f** (Figure 24 and Figure 25a).

The different behavior was analyzed by quantum chemical calculations (Figure 25b). In the same way that the nucleophilicity order of the carbenes (**41** ≈ **42** > **43**, Figure 23) parallels the order of the Lewis basicities (methyl cation affinities) of the model compounds (**49c** ≈ **49a** > **49b**, Figure 25b bottom), the nucleophilicity order of the deoxy Breslow intermediates (**45a** > **45b** > **45c**, Figure 25a) also mirrors the order of the proton affinities of the model compounds (**47a** > **47b** > **47c**, Figure 25b, top) [107].

A rationalization for the different sequence in the two series can be derived from the nucleus-independent chemical shifts (NICS) [109–111], which are considered to be a measure of aromaticity. In agreement with the almost equal lengths of the exocyclic C–C bonds in **45a** (136.1 pm) and **45c** (135.4 pm), as determined by X-ray crystallography, none of the two heterocyclic rings in **47a** and **47c** shows aromatic character (NICS(1)). However, while the electrophilic addition to the exocyclic double bond of **47a** yields the cyclic conjugated 6π system in **48a**, the analogous electrophilic addition to **47c** yields the nonaromatic amidinium ion **48c**. The high nucleophilicity of **45a**, which is mirrored by the high proton affinity of **47a**, can thus be explained by the gain of aromaticity during electrophilic attack. The same line of arguments can be used to rationalize the higher nucleophilicities and basicities of the triazoline derivatives **45b** and **47b**, respectively [107].

As the unsaturated carbenes **49a** and **49b** have already a similar aromatic character as the azolium ions **48a** and **48b** generated by protonation, unsaturated carbenes neither show higher basicity nor higher nucleophilicity than their saturated analogues [107].

**Figure 26:**
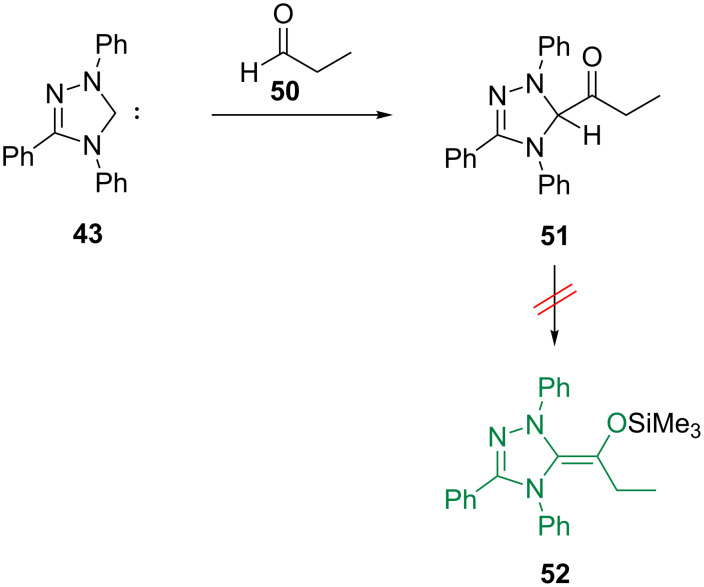
Berkessel’s synthesis of a Breslow intermediate (**51**, keto tautomer) from carbene **43** [112].

Are the properties of the deoxy Breslow intermediates also representative for the real Breslow intermediates? As shown by Berkessel and co-workers [112], Breslow intermediates generally exist as the keto tautomers **51**, and attempts to generate their O-silylated derivatives **52** have failed (Figure 26).

**Figure 27:**
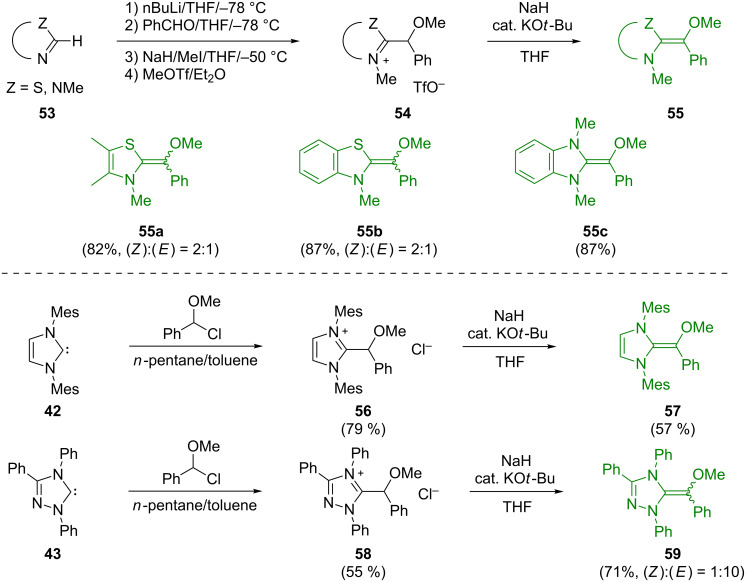
Synthesis of O-methylated Breslow intermediates [114].

In order to get closer to the actual Breslow intermediates than in Rovis’ aza-Breslow intermediates [113], we synthesized and isolated the O-methylated Breslow intermediates **55a**–**c**, **57**, and **59** as described in Figure 27 [114]. Some of them were characterized by single-crystal X-ray crystallography.

**Figure 28:**
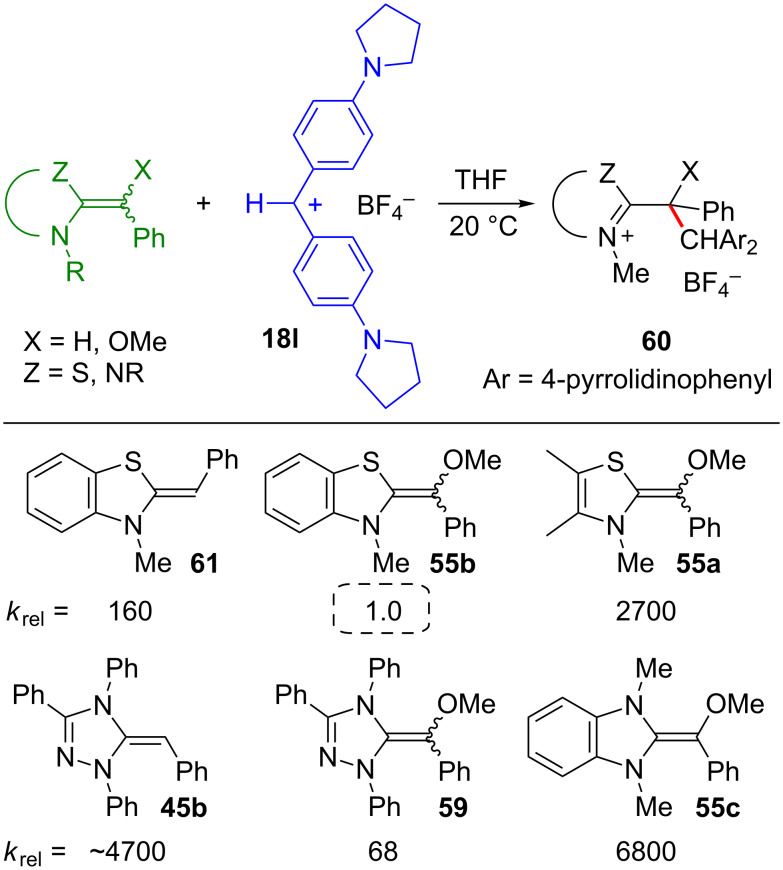
Relative reactivities of deoxy- and O-methylated Breslow intermediates [114].

Kinetic studies of their reactions with benzhydrylium ions provided their reactivity parameters *N* and *s*_N_ [114], and Figure 28 compares the relative reactivities of O-methylated and deoxy-Breslow intermediates toward the bis-pyrrolidino-substituted benzhydrylium ion **18l**. Comparison of the left and the central column shows that the O-methylated Breslow intermediates **55b** and **59** are 10^2^ times less reactive than their deoxy analogues **61** and **45b**, respectively. Obviously, the transition state is more affected by the destabilization of the cationic adduct due to the inductive electron-withdrawing effect than by the +M-effect of the methoxy group, which raises the HOMO of the reactants. Replacement of the sulfur atom in the benzothiazole by a NCH_3_ group (**55b** → **55c**) shows that imidazole derivatives are approximately four orders of magnitude more reactive than structurally analogous thiazole derivatives, which can, again, be assigned to the different electronegativities of sulfur and nitrogen.

## Conclusion

**Figure 29:**
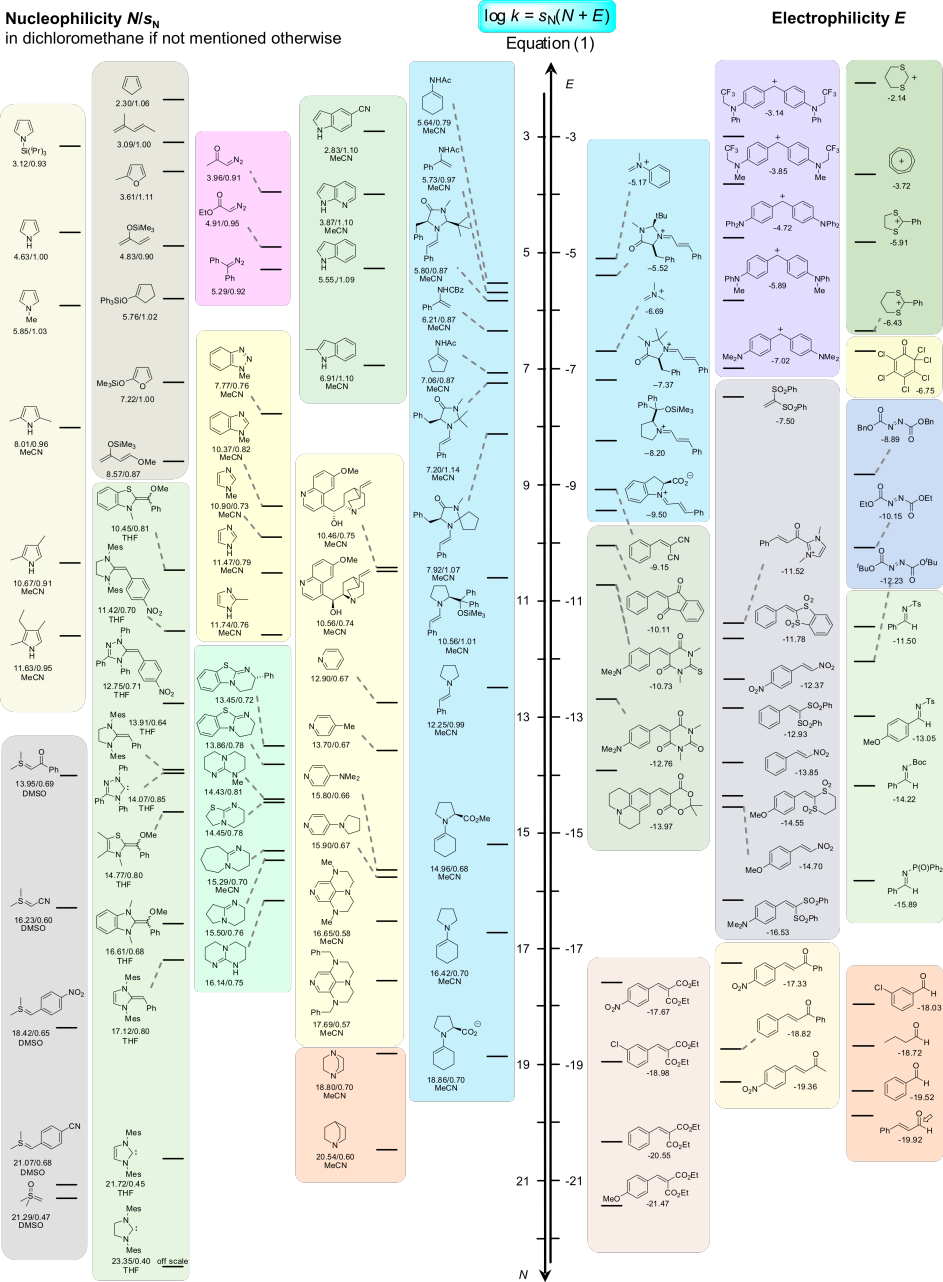
Reactivity scales for electrophiles and nucleophiles relevant for organocatalytic reactions (references and further reactivity parameters: [4]).

Organocatalytic reactions are complex multicomponent reactions, and a detailed description of the kinetics of the complete catalytic cycles is not yet possible. We have demonstrated, however, that important information can be obtained by specifically synthesizing relevant intermediates and studying the kinetics of their reactions with nucleophiles or electrophiles. By including them in our comprehensive electrophilicity and nucleophilicity scales (Figure 29), it has become possible to settle mechanistic controversies and to explore the scope of substrates suitable for iminium as well as for enamine activated reactions.

Rate and equilibrium studies of the reactions of N-heterocyclic carbenes and the corresponding deoxy Breslow intermediates showed that N-heterocyclic carbenes have similar nucleophilicities as other frequently employed organocatalysts, but are much stronger Lewis bases. The 10^3^ times higher nucleophilicities of benzylidene-imidazolines compared with benzylidene-imidazolidines explain why imidazol-2-ylidenes but not imidazolidine-2-ylidenes are commonly used organocatalysts.
